# Surgical aortic valve replacement etiologies, hemodynamics, and outcomes in 1346 patients from the Malaysian heart centre

**DOI:** 10.1186/s13019-023-02472-2

**Published:** 2024-01-02

**Authors:** Aslannif Roslan, Chong Kee Soon, Tey Yee Sin, Ahmad Tantawi Jauhari Aktifanus, Soh Si Ling, Wong Kian Boon, Beni I. Rusani, Hafidz Abd Hadi, Jayakhanthan Kolanthaivelu, Shaiful Azmi Yahaya, Jeswant Dillon, Alwi M. Yunus

**Affiliations:** Department of Cardiology and Department of Cardiothoracic Surgery, Institut Jantung Negara, 145, Jalan Tun Razak, 50400 Kuala Lumpur, Malaysia

**Keywords:** Echocardiography, Aortic valve, Prosthesis, Etiology, Low gradient

## Abstract

**Background:**

This study examined the characteristics and outcomes of surgical aortic valve replacement (SAVR) both isolated and in combination with other cardiac surgery in Malaysia from 2015 to 2021.

**Methods:**

This was a retrospective study of 1346 patients analyzed on the basis of medical records, echocardiograms and surgical reports. The overall sample was both considered as a whole and divided into aortic stenosis (AS)/aortic regurgitation (AR)-predominant and similar-severity subgroups.

**Results:**

The most common diagnosis was severe AS (34.6%), with the 3 most common etiologies being bicuspid valve degeneration (45.3%), trileaflet valve degeneration (36.3%) and rheumatic valve disease (12.2%). The second most common diagnosis was severe AR (25.5%), with the most common etiologies being root dilatation (21.0%), infective endocarditis (IE) (16.6%) and fused prolapse (12.2%). Rheumatic valve disease was the most common mixed disease. A total of 54.5% had AS-predominant pathology (3 most common etiologies: bicuspid valve degeneration valve, degenerative trileaflet valve and rheumatic valve disease), 36.9% had AR-predominant pathology (top etiologies: root dilatation, rheumatic valve disease and IE), and 8.6% had similar severity of AS and AR. Overall, 62.9% of patients had trileaflet valve morphology, 33.3% bicuspid, 0.6% unicuspid and 0.3% quadricuspid. For AS, the majority were high-gradient severe AS (49.9%), followed by normal-flow low-gradient (LG) severe AS (10.0%), paradoxical low-flow (LF)-LG severe AS (6.4%) and classical LF-LG severe AS (6.1%). The overall in-hospital and total 1-year mortality rates were 6.4% and 14.8%, respectively. Pure severe AS had the highest mortality. For AS-predominant pathology, the etiology with the highest mortality was trileaflet valve degeneration; for AR-predominant pathology, it was dissection. The overall survival probability at 5 years was 79.5% in all patients, 75.7% in the AS-predominant subgroup, 83.3% in the AR-predominant subgroup, and 87.3% in the similar-severity subgroup.

**Conclusions:**

The 3 most common causes of AS- predominant patients undergoing SAVR is bicuspid valve degeneration, degenerative trileaflet valve and rheumatic and for AR-predominant is root dilatation, rheumatic and IE. Rheumatic valve disease is an important etiology in our SAVR patients especially in mixed aortic valve disease.

*Study registration* IJNREC/562/2022.

## Background

Surgical aortic valve replacement (SAVR) is an established treatment for aortic valve dysfunction, be it aortic stenosis (AS), aortic regurgitation (AR) or a mixture of the two (mixed) [1]. There is a paucity of data on diagnosis, etiologies and outcomes in developing middle-income countries such as Malaysia. This is especially important for 2 reasons: (1) rheumatic valve disease (hereinafter, “rheumatic”) accounts for a substantial proportion of our SAVR population [2]; and (2) our SAVR population is mainly of Malay, Chinese and Indian ethnic backgrounds, and the etiologies and outcomes in this study might therefore be dissimilar to those in previous studies performed in Western countries. In addition, bicuspid aortic valve is the most common congenital cardiac condition [3], and its prevalence in Malaysian SAVR recipients is not known. Therefore, we sought to ascertain the following in all our SAVR recipients from 2015 to 2021: (1) the diagnosis leading to SAVR, (2) the etiology and outcomes of SAVR (both overall and divided into AS-predominant, AR-predominant, and similar-severity subgroups), (3) the prevalence and outcomes of various SAVR etiologies and (4) the prevalence and outcomes of low-gradient severe AS in our AS patients.

## Methods

### Patient population

**Fig. 1:**
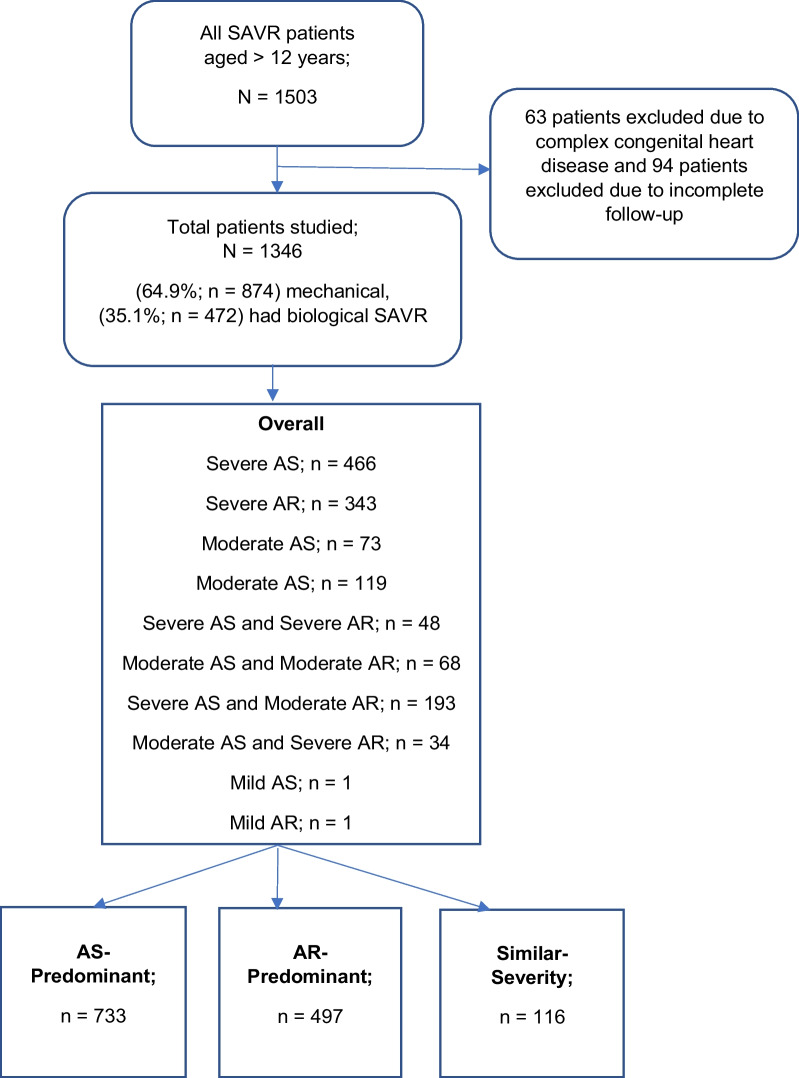
Flow chart in total, there were 1503 patients who underwent SAVR at IJN from 2016 to 2021. A total of 157 patients were excluded, and the other 1346 were analyzed. These patients were considered overall and subdivided into AS-predominant vs. AR-predominant vs. similar-severity subgroups

From the surgical registry, we analyzed all patients who underwent SAVR from 2015 to 2021 at the Institut Jantung Negara (IJN, National Heart Institute), which is Malaysia’s premier cardiac center and has the highest volume of cardiac surgeries for a single center in Southeast Asia (SEA). The study analyzed all SAVR recipients, including those who received isolated SAVR, double (mitral and aortic valve) replacement (DVR), SAVR and/or DVR with concomitant coronary artery bypass grafting (CABG), and SAVR with root replacement. We included all patients aged 12 years and above who did not have complex congenital heart disease. Both mechanical and biological SAVR are included as well (Fig. 1). This study was approved by the IJN (Institut Jantung Negara, Kuala Lumpur, Malaysia) ethics committee (IJNREC/562/2022).

Overall, 1503 patients underwent SAVR at IJN from 2015 to 2021. A total of 157 patients were excluded (63 patients excluded due to complex congenital heart disease and 94 excluded due to incomplete follow-up), and the other 1346 were analyzed. The included patients were considered overall and divided into AS-predominant, AR-predominant, and similar-severity subgroups. Mortality outcomes from both the diagnosis and diagnosis subgroups and for the different etiologies in the subgroups were analyzed.

### Diagnosis, mechanisms and hemodynamics

Diagnosis in this study refers to the type of dysfunction (stenosis vs. regurgitation vs. mixed) and its severity (mild vs. moderate vs. severe). Etiology refers to the mechanisms or causes of the dysfunction (bicuspid valve degeneration, trileaflet valve degeneration, rheumatic, dissection, etc.). The analysis was performed for a) the patients overall and b) the AS-predominant, AR-predominant, and similar-severity subgroups. For SAVR involving any degree of AS, we analyzed the echocardiographic features, particularly the aortic valve area and aortic valve area index (AVA and AVAi, respectively), mean pressure gradient across the aortic valve (meanPG) and stroke volume index (SVI), to classify them into high-gradient severe AS (HG-AS, meanPG > 40 mmHg, AVA < 1.0 cm^2^), classical low-flow low-gradient severe AS (CLF-LG-AS, meanPG < 40 mmHg, AVA < 1.0 cm^2^, EF < 50%, SVI < 35 ml/m^2^), paradoxical low-flow low-gradient severe AS (PLF-LG-AS, meanPG < 40 mmHg, AVA < 1.0 cm^2^, EF > 50%, SVI < 35 ml/m^2^), normal-flow low-gradient severe AS (NF-LG-AS, meanPG < 40 mmHg, AVA < 1.0 cm^2^, SVI > 35 ml/m^2^) and reverse area-gradient mismatch AS. Some terms that need further clarification are “degeneration”, which means calcification, thickening and/or retraction of leaflets causing stenosis and/or regurgitation, and “fused prolapse”, which means that the fused leaflets of the bicuspid aortic valve prolapse and cause regurgitation.

### Echocardiographic analysis

All preoperative transthoracic echocardiography (TTE) and intraoperative transesophageal echocardiography (TEE) recordings were analyzed by an expert echocardiographer specializing in valvular heart disease to determine the diagnosis, etiology and hemodynamics. Measurements of the aortic root (left ventricular outflow tract radius (LVOTr), sinus of Valsalva (SOV), sinotubular junction (STJ) and ascending aorta (AscAo)) and the number of leaflets (bicuspid vs. trileaflet vs. quadricuspid vs. unicuspid) were performed using TEE. The surgical reports where available was also analyzed to determine the number of leaflets but some of the reports does not have that information. Other pertinent echocardiographic parameters, such as biplane ejection fraction (EF), AVA, AVAi, peak velocity across the aortic valve (v max), meanPG, acceleration time (AT) and dimensionless velocity index (DVI), were analyzed using pre- and postoperative TTE. Preoperative TTE is echocardiography performed just before SAVR, and postoperative echocardiography is performed within 1 month after SAVR (if multiple scans were taken in that time, we considered only the first one after surgery).

### Electronic medical records (EMRs)

We extracted each patient’s age, gender and race from the EMRs. We analyzed in-hospital, 1-month, 6-month and 1-year mortality. Of these four variables, 1-month, 6-month, and 1-year mortality were defined to include only mortality after discharge from the hospital (i.e., in-hospital mortality was not included). We also calculated the *total* 1-year mortality, which was defined to include all in-hospital and post-discharge mortality in the first year. Finally, we performed Kaplan‒Meier analysis to obtain the 5-year probability of survival for our SAVR patients overall and for each of the groups (AS predominant vs. AR predominant vs. similar severity).

### Statistical analysis

Descriptive statistics were calculated to summarize the baseline demographic and clinical characteristics of the study patients. Continuous variables are summarized as the mean (M) with standard deviations (SD) or median with first and third quartiles (Q1 and Q3). Categorical variables are expressed as counts with percentages. The independent-samples *t*-test was carried out to compare the means of continuous variables. Differences among categorical groups were tested using the chi-square test or Fisher’s exact test as appropriate. Kaplan‒Meier analysis was used to predict the probability of all-cause mortality over the follow-up period.

## Results

This study included 1346 patients, of whom 534 (39.7%) were female and 812 (60.3%) were male. The mean age was 56.3 ± 14.9 years. A plurality of the patients was Malay (47.7%), followed by Chinese (29.6%), Indian (11.0%), other Malaysians (7.5%) and non-Malaysians (4.2%). The majority of patients underwent isolated AVR (50.7%, n = 683), followed by DVR (23.1%, n = 311) and AVR with CABG (16.3%, n = 219). A total of 64.9% (n = 874) had mechanical SAVR, and the rest (35.1%, n = 472) had biological SAVR.The high proportion of mechanical valves is due to the overall younger age of patients and a higher proportion of rheumatic valve disease in our SAVR population. The mean age for mechanical SAVR was 49.7 ± 12.9 years, while the mean age for biological SAVR was 68.6 ± 9.7 years. In terms of echocardiography parameters before and early after AVR, as expected, peak velocity decreased from 3.46 ± 1.39 m/s to 2.26 ± 0.54 m/s (*p* < 0.001), and meanPG decreased from 32.65 ± 25.42 mmHg to 11.14 ± 5.57 mmHg (*p* < 0.001). EF decreased slightly from 51.4 ± 11.54% to 47.34 ± 11.36% (*p* < 0.001) early after AVR (Table 1). The most common diagnosis was severe AS (34.6%), followed by severe AR (25.5%), severe AS combined with moderate AR (14.3%), and moderate AR (8.8%). The mean age of patients with severe AS was 63.4 ± 10.3 years, and that of patients with severe AR was 46.7 ± 16.0 years. For the 3 largest etiology groups of severe AS, the mean age for SAVR was 67.9 ± 7.7 years for trileaflet valve degeneration, 59.5 ± 10.0 years for bicuspid valve degeneration and 49.5 ± 14.3 years for rheumatic (Table 2).

**Table 1:** Demographics, procedure details and echocardiography

Variables (Total N = 1346)			n (%)
*Demographics*			
Gender		Female	534 (39.7)
		Male	812 (60.3)
Patient age		Mean (SD)	56.3 (14.9)
		Median (Q1, Q3)	59.6 (47.5, 67.0)
Ethnic group		Malay	642 (47.7)
		Chinese	399 (29.6)
		Indian	148 (11.0)
		Other Malaysians	101 (7.5)
		Foreigners	56 (4.2)
*Procedure details*			
Type of procedure		AVR	683 (50.7)
		DVR	311 (23.1)
		AVR and Root Replacement	78 (5.8)
		AVR and CABG	219 (16.3)
		AVR/Root Replacement/CABG	3 (0.2)
		CABG and DVR	24 (1.8)
		AVR and MV repair	19 (1.4)
		DVR and Root Replacement	4 (0.3)
		CABG and MV Repair and AVR	3 (0.2)
		AVR and TVR	1 (0.1)
		AVR and myectomy	1 (0.1)
Type valve replacement		Biological	472 (35.1)
		Mechanical	874 (64.9)
Patient age	Biological	Mean (SD)	68.6 (9.7)
		Median (Q1, Q3)	69.5 (65.9, 73.7)
	Mechanical	Mean (SD)	49.7 (12.9)
		Median (Q1, Q3)	52.5 (41.2, 59.8)

Echocardiography			
Variables	Mean (SD)		*p* Value
	Pre	Post	
SVI	49.37 (21.57)	39.16 (14.93)	< 0.001*
EF	51.4 (11.54)	47.34 (11.36)	< 0.001*
ET	0.32 (0.04)	0.24 (0.04)	< 0.001*
Flow rate	278.72 (128.13)	290.99 (111.79)	< 0.001*
Peak velocity	3.46 (1.39)	2.26 (0.54)	< 0.001*
MeanPG	32.65 (25.42)	11.14 (5.57)	< 0.001*
PeakPG	55.59 (40.16)	21.67 (10.62)	< 0.001*
DVI	0.37 (0.23)	0.52 (0.15)	< 0.001*
AVA	1.54 (1.3)	2.04 (0.91)	< 0.001*
AVAi	0.87 (0.72)	1.16 (0.51)	< 0.001*
AT	112.21 (28.03)	80.27 (17.13)	< 0.001*
AT/ET	0.36 (0.08)	0.34 (0.07)	< 0.001*

The majority of patients were male, and the most common ethnicity was Malay. The most common procedure was isolated AVR, followed by DVR and then AVR with CABG

**Table 2:** Diagnosis and etiology for SAVR patients

Diagnosis subgroup		Diagnosis			Etiology		
Variables	n (%) by total overall	Variables	n (%) by total diagnosis group	n (%) by total overall	Variables	n (%) by total diagnosis	n (%) by Total diagnosis group
AS Predominant	733 (54.5)	Severe AS	466 (63.6)	466 (34.6)	Bicuspid valve degeneration	211 (45.3)	211 (28.8)
					Trileaflet valve degeneration	169 (36.3)	169 (23.1)
					Rheumatic	57 (12.2)	57 (7.8)
					Unknown	24 (5.2)	24 (3.3)
					Unicuspid	4 (0.9)	4 (0.5)
					Radiation	1 (0.2)	1 (0.1)
					Total	466 (100.0)	
		Severe AS, Moderate AR	193 (26.3)	193 (14.3)	Rheumatic	67 (34.7)	67 (9.1)
					Bicuspid valve degeneration	60 (31.1)	60 (8.2)
					Trileaflet valve degeneration	55 (28.5)	55 (7.5)
					Unknown	6 (3.1)	6 (0.8)
					Radiation	2 (1.0)	2 (0.3)
					Degenerative and fused prolapse	1 (0.5)	1 (0.1)
					Fused prolapse	1 (0.5)	1 (0.1)
					Bicuspid valve degeneration and flail fused leaflets	1 (0.5)	1 (0.1)
					Total	193 (100.0)	
		Moderate AS	73 (10.0)	73 (5.4)	Bicuspid valve degeneration	29 (39.7)	29 (4.0)
					Trileaflet valve degeneration	21 (28.8)	21 (2.9)
					Rheumatic	19 (26.0)	19 (2.6)
					Unicuspid	2 (2.7)	2 (0.3)
					Unknown	1 (1.4)	1 (0.1)
					Rheumatic and bicuspid valve degeneration	1 (1.4)	1 (0.1)
		Mild AS	1 (0.1)	1 (0.1)	Bicuspid valve degeneration	1 (100.0)	1 (0.1)
					Total	1 (100.0)	
		Total	733 (100.0)		Total		733 (100.0)
AR Predominant	497 (36.9)	Severe AR	343 (69.1)	343 (25.5)	Root dilatation	72 (21.0)	72 (14.5)
					Infective endocarditis	57 (16.6)	57 (11.5)
					Fused prolapse	42 (12.2)	42 (8.5)
					Rheumatic	30 (8.7)	30 (6.0)
					Prolapsed RCC	26 (7.6)	26 (5.2)
					Unknown	18 (5.2)	18 (3.6)
					Dissection	17 (5.0)	17 (3.4)
					Prolapsed LCC	15 (4.4)	15 (3.0)
					Bicuspid valve degeneration	7 (2.0)	7 (1.4)
					SOV rupture	6 (1.7)	6 (1.2)
					Trileaflet valve degeneration	6 (1.7)	6 (1.2)
					Prolapsed NCC	6 (1.7)	6 (1.2)
					Flail RCC	5 (1.5)	5 (1.0)
					Flail fused	5 (1.5)	5 (1.0)
					Flail NCC	3 (0.9)	3 (0.6)
					Fused prolapse and root dilatation	3 (0.9)	3 (0.6)
					Perforation RCC	2 (0.6)	2 (0.4)
					Flail LCC	2 (0.6)	2 (0.4)
					Quadricuspid	2 (0.6)	2 (0.4)
					Prolapse of RCC into VSD	2 (0.6)	2 (0.4)
					Prolapsed NCC and root dilatation	2 (0.6)	2 (0.4)
					Destruction of LCC	1 (0.3)	1 (0.2)
					Root dilatation and fused prolapse	1 (0.3)	1 (0.2)
					Prolapsed LCC and root dilatation	1 (0.3)	1 (0.2)
					Prolapsed RCC and root dilatation	1 (0.3)	1 (0.2)
					Prolapsed NCC and RCC	1 (0.3)	1 (0.2)
					Partial fusion with large coaptation defect	1 (0.3)	1 (0.2)
					Retracted leaflets	1 (0.3)	1 (0.2)
					Prolapsed RCC and idiopathic aortic dilatation	1 (0.3)	1 (0.2)
					Post-myectomy complication	1 (0.3)	1 (0.2)
					Prolapsed RCC and root dilatation	1 (0.3)	1 (0.2)
					Root dilatation and prolapsed RCC	1 (0.3)	1 (0.2)
					Fused prolapse, root dilatation and bicuspid valve degeneration	1 (0.3)	1 (0.2)
					Flail NCC and root dilatation	1 (0.3)	1 (0.2)
					Flail RCC and root dilatation	1 (0.3)	1 (0.2)
					Retracted bicuspid	1 (0.3)	1 (0.2)
					Total	343 (100.0)	
		Moderate AR	119 (23.9)	119 (8.8)	Rheumatic	31 (26.1)	31 (6.2)
					Root dilatation	17 (14.3)	17 (3.4)
					Prolapsed RCC	16 (13.4)	16 (3.2)
					Trileaflet valve degeneration	16 (13.4)	16 (3.2)
					Fused prolapse	8 (6.7)	8 (1.6)
					Dissection	7 (5.9)	7 (1.4)
					Unknown	5 (4.2)	5 (1.0)
					Prolapsed LCC	4 (3.4)	4 (0.8)
					Bicuspid valve degeneration	4 (3.4)	4 (0.8)
					Infective endocarditis	3 (2.5)	3 (0.6)
					Prolapsed NCC	3 (2.5)	3 (0.6)
					Flail RCC	1 (0.8)	1 (0.2)
					Trileaflet valve degeneration and root dilatation with intramural hematoma	1 (0.8)	1 (0.2)
					Quadricuspid	1 (0.8)	1 (0.2)
					Radiation	1 (0.8)	1 (0.2)
					Degenerative due to subaortic membrane	1 (0.8)	1 (0.2)
					Total	119 (100.0)	
		Moderate AS, Severe AR	34 (6.8)	34 (2.5)	Rheumatic	12 (35.3)	12 (2.4)
					Fused prolapse	5 (14.7)	5 (1.0)
					Infective endocarditis	4 (11.8)	4 (0.8)
					Trileaflet valve degeneration	3 (8.8)	3 (0.6)
					Bicuspid valve degeneration	3 (8.8)	3 (0.6)
					Radiation	1 (2.9)	1 (0.6)
					Root dilatation and trileaflet valve degeneration	1 (2.9)	1 (0.6)
					Bicuspid valve degeneration and fused prolapse	1 (2.9)	1 (0.6)
					Prolapsed NCC	1 (2.9)	1 (0.6)
					Small LCC	1 (2.9)	1 (0.6)
					Prolapsed RCC	1 (2.9)	1 (0.6)
					Fused prolapse and annulus dilatation	1 (2.9)	1 (0.6)
					Total	34 (100.0)	
		Mild AR	1 (0.2)	1 (0.1)	Rheumatic	1 (100.0)	
					Total	1 (100.0)	
		Total	497 (100.0)		Total		497 (100.0)
Similar severity	116 (8.6)	Severe AS and Severe AR	48 (41.4)	48 (3.6)	Bicuspid valve degeneration	15 (31.3)	15 (12.9)
					Rheumatic	13 (27.1)	13 (11.2)
					Trileaflet valve degeneration	9 (18.8)	9 (7.8)
					Unknown	3 (6.3)	3 (2.6)
					Trileaflet valve degeneration and prolapsed NCC	1 (2.1)	1 (0.9)
					Bicuspid valve degeneration and fused prolapse	1 (2.1)	1 (0.9)
					Iatrogenic AR and degenerative AS	1 (2.1)	1 (0.9)
					Bicuspid valve degeneration and flail fused leaflets	1 (2.1)	1 (0.9)
					Subaortic membrane	1 (2.1)	1 (0.9)
					Flail fused and bicuspid valve degeneration	1 (2.1)	1 (0.9)
					Flail RCC	1 (2.1)	1 (0.9)
					Trileaflet valve degeneration and root dilatation	1 (2.1)	1 (0.9)
					Total	48 (100.0)	
		Moderate AS and Moderate AR	68 (58.6)	68 (5.1)	Rheumatic	33 (48.5)	33 (28.4)
					Trileaflet valve degeneration	17 (25.0)	17 (14.7)
					Bicuspid valve degeneration	10 (14.7)	10 (8.6)
					Radiation	1 (1.5)	1 (0.9)
					Quadricuspid	1 (1.5)	1 (0.9)
					Bicuspid valve degeneration and fused prolapse	1 (1.5)	1 (0.9)
					Bicuspid valve degeneration and root dilatation	1 (1.5)	1 (0.9)
					Unicuspid	1 (1.5)	1 (0.9)
					Unknown	1 (1.5)	1 (0.9)
					Fused prolapse	1 (1.5)	1 (0.9)
					Prolapsed LCC	1 (1.5)	1 (0.9)
					Total	68 (100.0)	
		Total	116 (100.0)		Total		116 (100.0)
Total	1346 (100.0)			1346 (100.0)			

Pure severe AS was the most common diagnosis, followed by pure severe AR. The most common etiology of pure severe AS was bicuspid valve degeneration, while that of pure AR was root dilatation. The most common diagnosis for AS predominant is bicuspid valve degeneration, and the most common diagnosis for AR predominant is root dilatation. Rheumatic is the most common diagnosis in the similar-severity group

The three most common causes of severe AS were bicuspid valve degeneration (45.3%, n = 211), followed by trileaflet valve degeneration (36.3%, n = 169) and rheumatic (12.2%, n = 57). There are many more varieties for severe AR. The most common etiology of severe AR was root dilatation (21.0%, n = 72) defined by any part of the aortic root with diameter > 40mm [4], followed by infective endocarditis (IE; 16.6%, n = 57), fused prolapse (12.2%, n = 42), rheumatic (8.7%, n = 30), prolapsed right coronary cusp (RCC; 7.6%, n = 26), unknown (5.2%, n = 18), dissection (5.0%, n = 17) and prolapsed left coronary cusp (LCC; 4.4%, n = 15). Rheumatic was the most common etiology for mixed diagnosis, commonest in moderate AS and moderate AR (48.5%, n = 33), severe AS and moderate AR (34.7%, n = 67), moderate AS and severe AR (35.3%, n = 12) and second most common for severe AS and severe AR (27.1%, n = 13). The most common diagnosis for mixed severe AS and severe AR was bicuspid valve degeneration (31.3%, n = 15). A complete list of the etiology for all the diagnoses is presented in Table 2.

**Fig. 2:**
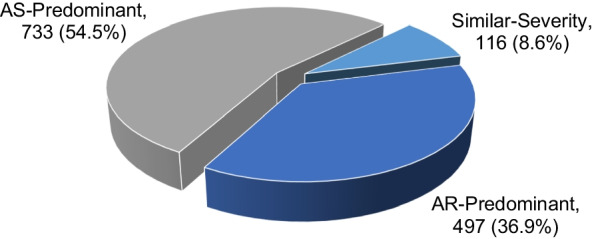
AS-predominant vs. AR-predominant vs. similar severity pathology. AS predominant was the most common pathology, followed by AR predominant and then similar severity

All diagnoses were then divided into AS-predominant (54.5%, n = 733), AR-predominant (36.9%, n = 497) and similar severity (8.6%, n = 116) Subgroup. (Figs. 1, 2 and Table 2). The most common cause of AS-predominant was bicuspid valve degeneration (41.1%, n = 301), followed by trileaflet valve degeneration (33.4%, n = 245) and rheumatic (19.5%, n = 143), which is similar to isolated severe AS. We had only 6 (0.8%) patients with unicuspid and 3 (0.4%) patients with radiation-induced AS. The most common etiology in the AR-predominant group was the same as that in the pure AR group, which was root dilatation (17.9%, n = 89), followed by rheumatic (14.9%, n = 74), infective endocarditis (12.9%, n = 64), fused prolapse (11.1%, n = 55), prolapsed RCC (8.7%, n = 43), trileaflet valve degeneration (5.0%, n = 25), dissection (4.8%, n = 24), prolapsed LCC (3.8%, n = 19), bicuspid valve degeneration (2.8%, n = 14), and prolapsed non-coronary cusp (NCC; 2%, n = 10). Finally, for similar severity, the most common etiology was rheumatic (39.7%, n = 46), followed by trileaflet valve degeneration (22.4%, n = 26) and bicuspid valve degeneration (21.6%, n = 25) (Table 2).

**Fig. 3:**
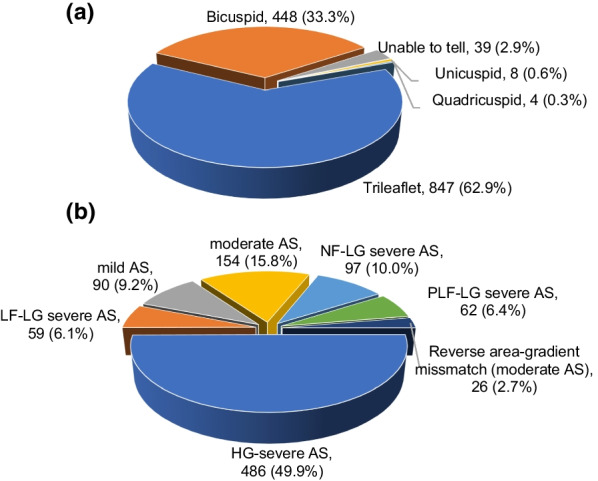
**a** Leaflet morphology. Leaflet morphology ascertained from TEE and/or surgical reports. The most common morphology was trileaflet (62.9%), followed by bicuspid (33.3%), unicuspid (0.6%) and quadricuspid (0.3%). In 2.9% of patients, the number of leaflets could not be determined. **b** AS classification, N = 974 Hemodynamics (gradient, flow and severity) for patients with any degree of AS. The most common category was HG-severe AS (49.9%), and the least common was reverse area-gradient mismatch (2.7%)

As expected, the majority of patients undergoing SAVR had trileaflet aortic valve morphology (62.9%, n = 847), followed by bicuspid (33.3%, n = 448), indeterminate (2.9%, n = 30), unicuspid (0.6%, n = 8) and quadricuspid (0.3%, n = 4) (Fig. 3a). Next, we analyzed the different hemodynamic profiles of aortic stenosis patients who underwent AVR. Of 1346 patients, 974 patients (72.4%) had some degree of aortic stenosis. The majority were HG-severe AS (49.9%, n = 486), followed by moderate AS (15.8%, n = 154), NF-LG severe AS (10.0%, n = 97), mild AS (9.2%, n = 90), PLF-LG severe AS (6.4%, n = 62), LF-LG severe AS (6.1%, n = 59) and reverse-area gradient mismatch (2.7%, n = 26) (Fig. 3b).

For all our SAVR patients, the mean inpatient stay was 11.7 ± 12.8 days. Overall in-hospital mortality was 6.4%. After discharge, the 1-month mortality was 2.6%, the 6-month mortality was 4.7%, and the 1-year mortality was 7.0%. The overall 1-year mortality, including in-hospital mortality, was 14.8%. In terms of mortality by specific diagnosis, pure severe AS had the highest in-hospital mortality (8.2%) and total 1-year mortality (including in-hospital mortality) (17.6%). For the grouping, in-hospital mortality was highest for AS-predominant (7.6%), followed by AR-predominant (5.0%) and finally similar severity (4.3%). After discharge, 1-year mortality (excluding in-hospital mortality) also follows a similar trend, with AS-predominant at 8.2%, AR-predominant at 6.1% and similar severity at 4.0%. The overall 1-year mortality (including in-hospital mortality) was 17.2% for AS-predominant patients, 12.1% for AR-predominant patients and 10.0% for patients with similar severity. For isolated SAVR, in-hospital mortality is lower at 5.6% vs. 6.4% for overall SAVR patients and total 1-year mortality is also lower at 14.1% vs 14.8% for overall SAVR patients (Table 3).

**Table 3:** Clinical outcomes

Inpatient stay		
Length of inpatient stay (days)	Mean (SD)	11.7 (12.8)
	Median (Q1, Q3)	8.0 (7.0, 12.0)

All-cause mortality rate					
Variables	In-Hospital; n (%)	Follow-up; n (%)			Total 1-year; n (%)
		1-month	6-month	1-year	
	N = 1346	N = 1128	N = 1033	N = 951	N = 1037
*Overall*					
Total mortality	86 (6.4)	29 (2.6)	49 (4.7)	67 (7.0)	153 (14.8)
*AVR Group*					
Isolated AVR mortality	38 (5.6)	15 (2.6)	25 (4.7)	35 (7.3)	73 (14.1)
Non-isolated AVR	48 (7.2)	14 (2.6)	24 (4.8)	32 (6.8)	80 (15.4)
Diagnosis					
Severe AS	38 (8.2)	10 (2.6)	18 (5.0)	26 (8.0)	64 (17.6)
Severe AR	20 (5.8)	9 (3.1)	12 (4.4)	15 (5.9)	35 (12.7)
Moderate AS	5 (6.8)	1 (1.6)	4 (6.8)	5 (9.4)	10 (17.2)
Moderate AR	4 (3.4)	1 (1.0)	4 (4.4)	5 (6.0)	9 (10.3)
Severe AS and Severe AR	2 (4.2)	1 (2.4)	1 (2.7)	2 (5.4)	4 (10.3)
Moderate AS and Moderate AR	3 (4.4)	0	1 (2.3)	1 (2.6)	4 (9.8)
Severe AS, Moderate AR	13 (6.7)	6 (3.8)	7 (4.8)	11 (8.3)	24 (16.4)
Moderate AS, Severe AR	1 (2.9)	1 (3.4)	2 (7.7)	2 (8.0)	3 (11.5)
Mild AS	0	0	0	0	0
Mild AR	0	0	0	0	0
*AS-Predominant vs. AR-Predominant vs. Similar-Severity Group*					
AS Predominant	56 (7.6)	17 (2.8)	29 (5.1)	42 (8.2)	98 (17.2)
AR Predominant	25 (5.0)	11 (2.6)	18 (4.7)	22 (6.1)	47 (12.1)
Similar Severity	5 (4.3)	1 (1.1)	2 (2.5)	3 (4.0)	8 (10.0)
*AS-Predominant by Etiology*					
Bicuspid valve degeneration	14 (4.7)	3 (1.1)	5 (2.0)	6 (2.7)	20 (8.5)
Trileaflet valve degeneration	33 (13.5)	7 (3.5)	15 (8.1)	23 (13.4)	56 (27.3)
Rheumatic	6 (4.2)	4 (3.6)	6 (6.0)	9 (10.1)	15 (15.8)
Unicuspid	0	0	0	0	0
Radiation	0	0	0	0	0
Others	3 (8.6)	3 (10.0)	3 (10.7)	4 (16.0)	7 (25.0)
*AR-Predominant by Etiology*					
Root dilatation	5 (5.6)	2 (2.6)	5 (7.4)	5 (7.8)	10 (14.5)
Rheumatic	1 (1.4)	0	2 (3.8)	2 (3.9)	3 (5.8)
Infective endocarditis	7 (10.9)	2 (4.2)	2 (4.7)	3 (7.1)	10 (20.4)
Fused prolapse	1 (1.8)	1 (2.1)	1 (2.2)	1 (2.6)	2 (5.0)
Prolapsed RCC	2 (4.7)	0	2 (5.3)	3 (8.3)	5 (13.2)
Trileaflet valve degeneration	2 (8.0)	1 (4.5)	1 (5.3)	2 (11.1)	4 (20.0)
Dissection	3 (12.5)	1 (5.0)	1 (5.9)	2 (11.8)	5 (25.0)
Prolapsed LCC	1 (5.3)	2 (11.8)	2 (11.8)	2 (12.5)	3 (17.6)
Bicuspid valve degeneration	0	0	0	0	0
Prolapsed NCC	0	0	0	0	0
Flail RCC	1 (16.7)	0	0	0	1 (33.3)
SOV rupture	0	0	0	0	0
Flail fused	0	0	0	0	0
Flail NCC	1 (33.3)	0	0	0	1 (33.3)
Quadricuspid	0	0	0	0	0
Fused prolapse and root dilatation	0	0	0	0	0
Others	1 (1.9)	2 (4.4)	2 (4.7)	2 (5.0)	3 (7.3)
*Similar-Severity by Etiology*					
Rheumatic	1 (2.2)	1 (2.9)	1 (3.3)	2 (8.0)	3 (11.5)
Trileaflet valve degeneration	2 (7.7)	0	0	0	2 (10.0)
Bicuspid valve degeneration	1 (4.0)	0	0	0	1 (5.9)
Others	1 (5.3)	0	1 (6.3)	1 (6.3)	2 (11.8)
*AS Hemodynamic*					
HG-severe AS	29 (6.0)	11 (2.7)	14 (3.8)	23 (6.8)	52 (14.2)
PLF-LG severe AS	7 (11.3)	1 (2.0)	3 (6.3)	5 (11.6)	12 (24.0)
NF-LG severe AS	8 (8.2)	2 (2.4)	4 (4.9)	6 (7.9)	14 (16.7)
LF-LG severe AS	8 (13.6)	3 (6.7)	5 (11.9)	5 (13.5)	13 (28.9)
Moderate AS	8 (5.2)	2 (1.6)	6 (5.2)	7 (6.6)	15 (13.2)
Mild AS	6 (6.7)	1 (1.4)	2 (3.2)	2 (3.5)	8 (12.7)
Reverse area-gradient mismatch (moderate AS)	1 (3.8)	0	1 (5.3)	1 (6.7)	2 (12.5)

The overall in-hospital and total 1-year mortality rates were 6.4% and 14.8%, respectively. Pure severe AS has the highest in-hospital and total 1-year mortality. Bicuspid valve degeneration has had the best survival at 1 year for AS-predominant etiology, followed by rheumatic, and the worst is trileaflet valve degeneration. Dissection and IE have the worst outcome for AR predominant subgroup. Hemodynamically for AS, the poorest outcome occurs in LF-LG severe AS

In-hospital” indicates those patients who died in-hospital before discharge

Follow-up” indicates those patients who were alive at discharge but died at 1-month, 6-months and 1-year follow-up

Total 1-year” indicates total number of patients who are dead at 1-year including in-hospital deaths

**Fig. 4:**
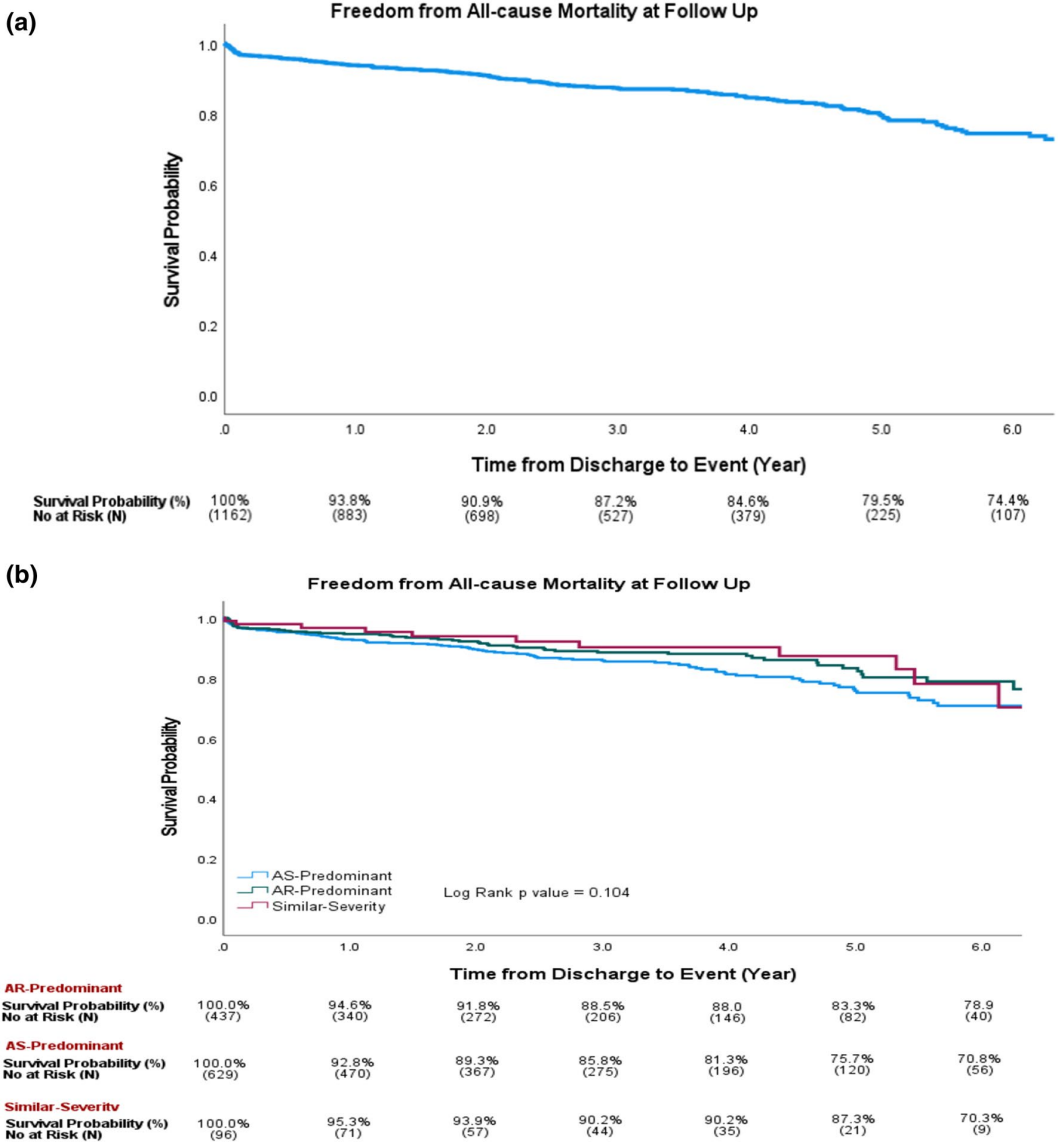
**a** Kaplan‒Meier curves for patients overall For all SAVR patients, the probability of survival at 5 years was 79.5%. **b** Kaplan‒Meier curves for the AS-predominant, AR-predominant, and similar-severity groups. For the AS-predominant group, the probability of 5-year survival was 75.7%; for the AR-predominant group, it was 83.3%; and for the similar-severity group, it was 87.3%

For patients with predominant AS, the etiology with the highest in-hospital mortality was trileaflet valve degeneration (13.5%), followed by bicuspid valve degeneration (4.7%) and rheumatic (4.2%). The highest total 1-year mortality (including in-hospital mortality) was for trileaflet valve degeneration (27.3%), followed by rheumatic (15.8%) and bicuspid valve degeneration (8.5%). For patients with AR-predominant, excluding flail RCC and flail NCC due to very small number of patients, the highest in-hospital mortality is dissection (12.5%), followed by infective endocarditis (10.9%). The highest total 1-year mortality (including in-hospital mortality) was also for dissection (25.0%), followed by infective endocarditis (20.4%). For the similar severity group, the highest in-hospital mortality was for trileaflet valve degeneration (7.7%). For different types of aortic stenosis hemodynamics (excluding mild AS), in-hospital mortality was highest for LF-LG severe AS (13.6%), followed by PLF-LG severe AS (11.3%), NF-LG severe AS (8.2%), HG-severe AS (6.0%), moderate AS (5.2%) and reverse-area gradient mismatch (3.8%). For overall 1-year mortality (including in-hospital mortality), the highest was still LF-LG severe AS (28.9%), followed by PLF-LG severe AS (24.0%), NF-LG severe AS (16.7%), HG-severe AS (14.2%), moderate AS (13.2%) and reverse area-gradient mismatch (12.5%) (Table 3). The overall survival probability at 5 years was 79.5% in all patients, 75.7% in the AS-predominant subgroup, 83.3% in the AR-predominant subgroup, and 87.3% in the similar-severity subgroup (Fig. 4a and b).

## Discussion

In this study, severe AS (34.6%) was the most common diagnosis, followed by severe AR (25.5%). In-hospital mortality is 6.4% and total 1-year mortality is 14.8% for overall SAVR patients and 5.6% (in-hospital) and 14.1% (total 1-year) for isolated SAVR patients. SAVR has been the mainstay of treatment for aortic valve dysfunction for more than 50 years since the first aortic valve replacement was performed in 1962 [1]. SAVR has been successful, with an overall mortality rate of 2.5% for AS and less than 1% for asymptomatic severe AS in patients < 70 years old [5]. As far as we are aware, there are no publications looking at etiology and outcome of SAVR in developing countries such as Malaysia. This is important, as the etiology may be different with more rheumatic heart disease and different racial and genetic make-up of our populations [6, 7]. In this study, patients with pure severe AS underwent SAVR at a later age (63.4 ± 10.3 years) than those with severe AR (46.7 ± 16.0 years). For severe AS, this is somewhat similar to the landmark Braunwald circulation paper in 1968, where the onset of symptoms was at 60 years old, whereas now in Western societies, it is at 75 years old due to the lower prevalence of rheumatic heart disease [8].

For isolated severe AS, we had more patients with bicuspid valve degeneration (45.3%) compared to trileaflet valve degeneration (36.3%). A previous study in a Western population also showed slightly more bicuspid (49%) vs. trileaflet (46%) aortic valves [9]. Rheumatic form a larger proportion compared to those from Western countries for both isolated severe AS (12.2%, 3rd most common) and even more so for AS predominant (19.5%, 3rd most common) [9, 10]. In fact, when we look at mixed aortic valve diseases, the most common etiology is rheumatic valve disease, especially when dealing with similar severity (moderate AS/AR, severe AS/AR). For AR, the etiology is much more diverse, with the most common cause being root dilatation (21%), followed by IE (16.6%) and fused prolapse (12.2%). Compared with contemporary data from the Mayo Clinic, cusp prolapse is the most common diagnosis, followed by degenerative and root dilatation. Their paper, however, did not separate fused prolapse (bicuspid) with single leaflet prolapse, whereas in this study, the frequency of each type of leaflet prolapse was ascertained [11].

As expected when we considered SAVR recipients overall as opposed to those with severe AS or AS-predominant pathology only, the majority had trileaflet valves (62.9%, n = 847), followed by bicuspid valves (33.3%, n = 448). This study had a higher percentage of bicuspid valves (33.3% vs. 23.0%) and a lower percentage of trileaflet valves (62.9% vs. 77.0%) than the 2020 study of SAVR in the Swedish population [12]. Low gradient severe aortic stenosis may arise in patients with inherently small aortic annuli or low forward stroke volume due to poor systolic function, high arterial afterload, or small left ventricular chamber size [13]. In this study, looking at severe AS (isolated or mixed), 31% (218/704) had low-gradient severe AS, which is less than the most recent study in Australia showing that half of severe AS cases have low-gradient hemodynamics [14]. This is most likely due to less recognition and treatment for this confusing entity. The most recent entity recognized [15], reverse area-gradient mismatch, is the least common at 2.7%.

Overall in-hospital mortality was 6.4%. After discharge, the 6-month mortality was 4.7%, and the 1-year mortality (excluding in-hospital) was 7.0%. This appears higher than the Swedish study (30-day mortality = 2.3% and 1 year = 3.4%) [12]. Possible reasons for this are later presentation, waiting too long before intervention, less access to and awareness of healthcare with less than 10 government hospitals that is able to provide cardiothoracic surgery services. Patients with pure severe AS had the highest inpatient and total 1-year mortality (8.2% and 17.6%, respectively). The same was also true with AS-predominant having higher mortality compared to AR-predominant and similar severity. Trileaflet valve degeneration was the etiology with the highest in-hospital and total mortality for AS-predominant patients (13.5% and 27.3%, respectively), most likely because this cohort of patients had the most advanced age at surgery. For both isolated severe AS and AS-predominant, the bicuspid has much better survival similar to study done in developed country [12]. The total 1-year survival after SAVR for AS-predominant subgroup for rheumatic aortic stenosis (15.8%) is between trileaflet valve degeneration (27.3%) and bicuspid valve degeneration (8.5%).

In AR-predominant patients, the etiology with the highest in-hospital and total mortality was dissection (12.5% and 25.0%, respectively), followed by IE (10.9% and 20.4%, respectively). This is not surprising, as both of these conditions, even with modern treatment, are still very dangerous, as shown by a study in Finland after surgery for type A dissection (30-day mortality = 15.7%) [16] and another study by Nguyen et al. [17] showing high 5-year mortality after SAVR for IE. In terms of different severe aortic stenosis hemodynamics, the highest in-hospital and total 1-year mortality occurred in LF-LG severe AS (13.6%, 28.9%), and the lowest occurred in straightforward HG-severe AS (6.0%, 14.2%). LF-LG severe AS also had the poorest total 1-year mortality in an Australian study (30.5%), but in their study, NF-LG severe AS had the best total 1-year mortality (11.6%) [14]. Reverse area-gradient mismatch had the best in-hospital and total 1-year mortality (3.8% and 12.5%, respectively) in this study. To the best of our knowledge, there are no studies on SAVR outcomes in this clinical entity.

### Study limitations

This is a retrospective study and we cannot control for selection bias, confounding variables and generalizability. This study involved all patients who underwent SAVR regardless of concomitant mitral bypass grafts or root replacements; these concomitant surgeries can also affect a patient’s outcome. Second, the EMR available in our center is still noncomprehensive, such that each patient’s comorbidities could not be analyzed in detail; this shortcoming could also affect the study results.

## Conclusions

The most common SAVR diagnosis was severe AS, followed by severe AR. Bicuspid valve degeneration, trileaflet valve degeneration and rheumatic were the 3 most common etiologies for both severe AS and AS-predominant patients. Root dilatation was the most common cause of severe AR. In-hospital mortality is 6.4% and total 1-year mortality is 14.8% for overall SAVR patients and 5.6% (in-hospital) and 14.1% (total 1-year) for isolated SAVR patients. Isolated severe AS, especially LF-LG severe AS, had the highest in-hospital and overall 1-year mortality.

## Data Availability

All the data will be made available upon written requests.

## References

[1] Lim JCES, Elliott MJ, Wallwork J, Keogh B (2022). Cardiac surgery and congenital heart disease: reflections on a modern revolution. Heart.

[2] Liang-Choo H, Rajaram N (2016). A review of acute rheumatic fever and rheumatic heart disease research in Malaysia. Med J Malaysia.

[3] Durán AC, Daliento L, Frescura C, Stellin G, Sans-Coma V, Angelini A (1995). Unicommissural aortic valve in neonates and its association with other congenital heart disease. Cardiol Young.

[4] Pelliccia A, Di Paolo FM, De Blasiis E, Quattrini FM, Pisicchio C, Guerra E (2010). Prevalence and clinical significance of aortic root dilation in highly trained competitive athletes. Circulation.

[5] Carabello BA (2013). Introduction to aortic stenosis. Circul Res.

[6] Sliwa K, Carrington M, Mayosi BM, Zigiriadis E, Mvungi R, Stewart S (2010). Incidence and characteristics of newly diagnosed rheumatic heart disease in Urban African adults: Insights from the Heart of Soweto Study. Eur Heart J.

[7] Khoo PLZ, Poon JS, Tan GJS, Awang Y, Chan KMJ (2020). A review of heart valve disease research in Malaysia. Med J Malays.

[8] Ross J, Braunwald E (1968). Aortic stenosis. Circulation.

[9] Roberts WC, Ko JM (2005). Frequency by decades of unicuspid, bicuspid, and tricuspid aortic valves in adults having isolated aortic valve replacement for aortic stenosis, with or without associated aortic regurgitation. Circulation.

[10] Czarny MJ, Resar JR (2014). Diagnosis and management of valvular aortic stenosis. Clin Med Insights Cardiol.

[11] Yang LT, Michelena HI, Maleszewski JJ, Schaff HV, Pellikka PA (2019). Contemporary etiologies, mechanisms, and surgical approaches in pure native aortic regurgitation. Mayo Clin Proc..

[12] Holmgren A, Enger TB, Näslund U, Videm V, Valle S, Evjemo KJD (2021). Long-term results after aortic valve replacement for bicuspid or tricuspid valve morphology in a Swedish population. Eur J Cardio-thoracic Surg.

[13] Eleid MF, Nishimura RA, Borlaug BA, Sorajja P (2013). Invasive measures of afterload in low gradient severe aortic stenosis with preserved ejection fraction. Circ Heart Fail.

[14] Snir AD, Ng MK, Strange G, Playford D, Stewart S, Celermajer DS (2021). Prevalence and outcomes of low-gradient severe aortic stenosis—from the national echo database of Australia. J Am Heart Assoc.

[15] Abbas AE, Pibarot P (2019). Hemodynamic characterization of aortic stenosis states. Catheterizat Cardiovascul Intervent.

[16] Jormalainen M, Kesävuori R, Raivio P, Vento A, Mustonen C, Honkanen HP (2022). Long-term outcomes after ascending aortic replacement and aortic root replacement for type A aortic dissection. Interact Cardiovasc Thorac Surg.

[17] Nguyen DT, Delahaye F, Obadia JF, Duval X, Selton-Suty C, Carteaux JP (2010). Aortic valve replacement for active infective endocarditis: 5-year survival comparison of bioprostheses, homografts and mechanical prostheses. Eur J Cardio-thoracic Surg.

